# Impact of antibiotic perturbation on fecal viral communities in mice

**DOI:** 10.1093/g3journal/jkac293

**Published:** 2022-11-22

**Authors:** Jacqueline Moltzau Anderson, Tim Lachnit, Simone Lipinski, Maren Falk-Paulsen, Philip Rosenstiel

**Affiliations:** Institute of Clinical Molecular Biology, Christian-Albrechts University of Kiel, 24098 Kiel, Germany; Zoological Institute, Christian-Albrechts University of Kiel, 24098 Kiel, Germany; Institute of Clinical Molecular Biology, Christian-Albrechts University of Kiel, 24098 Kiel, Germany; Institute of Clinical Molecular Biology, Christian-Albrechts University of Kiel, 24098 Kiel, Germany; Institute of Clinical Molecular Biology, Christian-Albrechts University of Kiel, 24098 Kiel, Germany

**Keywords:** virome, intestinal bacteriophages, antibiotic perturbation, NOD2, resilience

## Abstract

Viruses and bacteriophages have a strong impact on intestinal barrier function and the composition and functional properties of commensal bacterial communities. Shifts of the fecal virome might be involved in human diseases, including inflammatory bowel disease (IBD). Loss-of-function variants in the nucleotide-binding oligomerization domain-containing protein 2 (*NOD2*) gene are associated with an increased risk of developing Crohn’s disease, a subtype of human chronic IBD, where specific changes in fecal viral communities have also been described. To improve our understanding of the dynamics of the enteric virome, we longitudinally characterized the virome in fecal samples from wild-type *C57BL/6J* and *NOD2* knock-out mice in response to an antibiotic perturbation. Sequencing of virus-like particles demonstrated both a high diversity and high interindividual variation of the murine fecal virome composed of eukaryotic viruses and bacteriophages. Antibiotics had a significant impact on the fecal murine virome. Viral community composition only partially recovered in the observation period (10 weeks after cessation of antibiotics) irrespective of genotype. However, compositional shifts in the virome and bacteriome were highly correlated, suggesting that the loss of specific phages may contribute to prolonged dysregulation of the bacterial community composition. We suggest that therapeutic interference with the fecal virome may represent a novel approach in microbiota-targeted therapies.

## Introduction

Viruses are an integral part of the gut microbiome (Reyes *et al.* 2010; Minot *et al.* 2011). Mostly comprised of bacteriophages, this community is temporally stable in the absence of disease (Reyes *et al.* 2010; Minot *et al.* 2011). Alterations in the gut microbiome have been associated with inflammatory bowel disease (IBD), a human relapsing chronic inflammatory disease, for which the precise cause remains unknown. Evidence suggests that environmental factors, including a Western industrialized lifestyle may lead to shifts in the microbiota, which contribute to the anomalous intestinal inflammatory response in genetically susceptible hosts (Chang 2020). The first Crohn’s disease susceptibility gene nucleotide-binding and oligomerization domain 2 (*NOD2*) represents a cornerstone of this concept. Physiologically, *NOD2* is an intracellular sensor of muramyl dipeptide, a peptidoglycan fragment (Girardin *et al.* 2003; Inohara *et al.* 2003). It is involved in surveillance and control of the intestinal microbiota as it orchestrates the expression of several barrier-protective molecules such as defensins, DMBT1 and reactive oxygen species (Wehkamp *et al.* 2004; Kobayashi *et al.* 2005; Rosenstiel *et al.* 2007; Lipinski *et al.* 2009). Several studies suggest that *NOD2* may be also involved in recognizing viral infections (Sabbah *et al.* 2009; Lupfer *et al.* 2013). Genetic loss-of-function variants in its sensor domain are associated with increased susceptibility for Crohn’s disease (Hugot *et al.* 2001; Ogura *et al.* 2001).

Previously, we demonstrated evidence for the role of *NOD2* in controlling resilience of the intestinal microbiota (bacteriome and mycobiome), whereby the impaired recovery dynamics of the microbiota after antibiotic perturbation in *NOD2*-deficient mice is contributing to an inflammation-prone state of the intestinal mucosa (Moltzau Anderson *et al.* 2020). Such alteration in the capacity to restore a physiological equilibrium (i.e. long-term community stability and stable ecosystem services supporting a healthy human physiology) could be involved in the etiology of chronic inflammatory diseases and other intestinal disorders (Moltzau Anderson *et al.* 2020). In favor of this hypothesis, it has been demonstrated in several human cohort studies that diversity and functional properties of the intestinal microbiota of IBD patients display higher temporal fluctuation compared with healthy subjects, indicating a potential loss of host homeostasis (Ott *et al.* 2004; Manichanh *et al.* 2006; Frank *et al.* 2007; Willing *et al.* 2010).

Here, we hypothesized that the absence of functional *NOD2* may have a direct influence on the intestinal virome. In contrast to the bacteriome, little is known about the fecal viral communities in response to a specific pulse perturbation, in which an external event causes a distinct selective pressure on the intestinal ecosystem for a short period of time (i.e. a short course of antibiotics) (Sommer *et al.* 2017). Phages have been found to have various effects on the bacterial community, by impacting bacterial diversity in a community, stimulating evolutionary change, and providing selective advantages to their bacterial hosts (Abeles *et al.* 2015). Although it is obvious that shifts of bacterial taxa by specific antibiotics will directly cause secondary changes of the intestinal virome composition, it is likely that residing bacteriophages may exert an important level of control on the dynamics of bacterial community recovery by negative selection (Abeles *et al.* 2015). Moreover, an enteric eukaryotic virus, murine norovirus, was shown to replace the beneficial function of the commensal bacteria by offsetting the effect of treatment with antibiotics in germ-free and antibiotic treated mice (Kernbauer *et al.* 2014). Thus, viruses can also play an important role in the regulation of intestinal homeostasis in response to antibiotic perturbations.

Viruses have also been shown to be extremely diverse, varying in their genetic material, genome sizes, life cycles, transmission routes, or persistence (Sharp 2002; Colson *et al.* 2013; Popgeorgiev *et al.* 2013; Colson *et al.* 2017). Humans are colonized by large populations of viruses consisting of viruses that infect eukaryotic cells (eukaryotic viruses) and those that infect bacteria (bacteriophages) (Popgeorgiev *et al.* 2013; Handley 2016). Human feces are estimated to contain at least 10^9^ virus-like particles (VLPs) per gram (Minot *et al.* 2013), and although many of these viruses have been identified as bacteriophages, the majority remain unidentified (Reyes *et al.* 2010, 2012; Minot *et al.* 2011). Furthermore, genomes of fecal bacteria harbor virus-derived genetic elements (e.g. prophages and retroviral elements), which might be activated and may contribute to further complexity (Belshaw *et al.* 2004; Popgeorgiev *et al.* 2013; Handley 2016). Metagenomic analyses of human gut viruses have revealed extreme interindividual diversity (i.e. diversity between individuals). This is in part likely due to the already considerable individual variation in the bacterial strains present in the gut, for which differences in phage predators are influenced (Rodriguez-Valera *et al.* 2009; Minot *et al.* 2011). It is well established that phages can be highly selective for different bacteria, and as such, phage sensitivity (phage typing) has been used for decades as an effective means of differentiating between different bacterial strains (Sell *et al.* 1983; Schofield *et al.* 2012). Rapid within-host viral evolution may also influence the large variability among individuals. In a long-term study investigating the viral community of an adult individual, Microviridae, a family of bacteriophages, was demonstrated to have high substitution rates, causing the sequence divergence values to be sufficient to distinguish new viral species by the conclusion of the study (Minot *et al.* 2013). Moreover, individual virome compositions has been suggested to be relatively stable, with an estimated 80% of viral forms to be persistent throughout a 2.5-year-long study (Minot *et al.* 2013), with similar findings also observed in studies of shorter duration (Reyes *et al.* 2010; Minot *et al.* 2011).

**Fig. 1.**
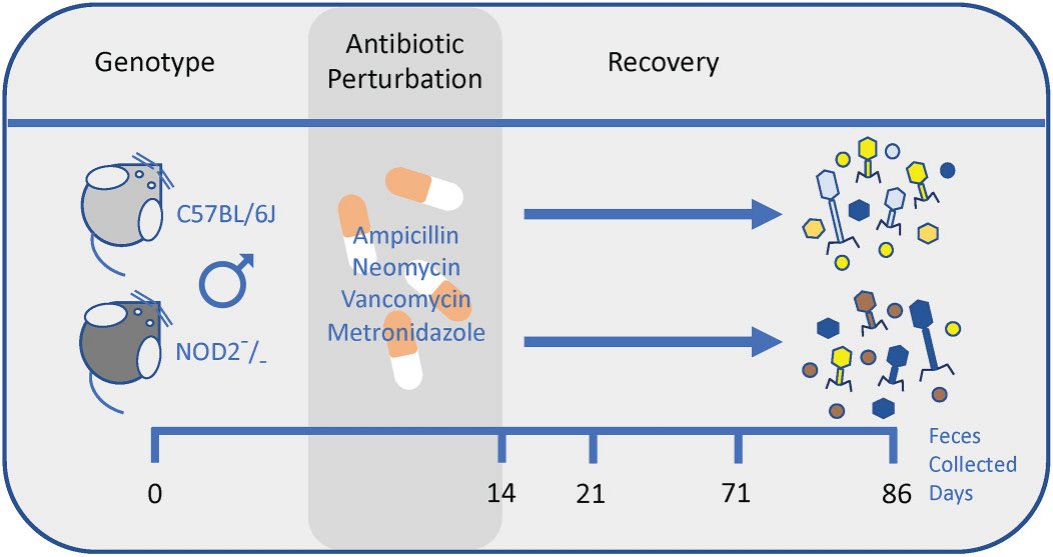
Study design demonstrating *NOD2* KO (*n* = 3) and *C57BL/6J* WT (*n* = 3) male mice perturbed by a treatment with a combination of antibiotics (ampicillin, neomycin, vancomycin, and metronidazole) for 12 days (days 2–14). Mice were subsequently monitored for a total of 86 days to investigate viral community recovery (post day 14). Fecal samples were collected on day 0 (pre-treatment), day 14 (during treatment), and days 21, 71 and 86 (recovery period). Previously, we investigated the bacterial and fungal communities in this same model (Moltzau Anderson *et al.* 2020).

Here, we investigated the effect of an antibiotic perturbation on the longitudinal variation of fecal viral communities in *C57BL/6J* wild-type (WT) and *NOD2* knock-out (KO) mice (Fig. 1) (Moltzau Anderson *et al.* 2020). Elucidating such defined temporal variation of the fecal virome might be critical toward understanding the role of this insufficiently described layer of the microbiota and its impact on gut health.

## Materials and methods

### Animals

All animal experiments were approved by the local animal safety review board of the federal ministry of Schleswig Holstein and conducted according to national and international laws and policies [V 312-72241.121-33 (95-8/11)]. All animals were housed in a mouse facility at the Christian-Albrechts University of Kiel and experiments carried out as previously described (Moltzau Anderson *et al.* 2020). Briefly, a single *NOD2*-deficient male mouse was crossed with a *C57BL/6J* female to obtain heterozygous offspring (F1), from which WT and *NOD2* KO breeder pairs were generated (F2). Male offspring of the next 2 generations were then used and maintained in single cages under specific-pathogen free conditions. At the onset of the study (day 0), mice were approximately 52 weeks old. To cause a significant perturbation on the gut microbiome of the mice, we used a variation of the commensal depletion protocol by treating *C57BL/6J* WT and *NOD2* KO male mice for 2 weeks with broad-spectrum antibiotics composed of ampicillin (1 g/l), vancomycin (500 mg/l), neomycin (1 g/l), and metronidazole (1 g/l) (Sigma-Aldrich) (Fagarasan *et al.* 2002; Rakoff-Nahoum *et al.* 2004; Vijay-Kumar *et al.* 2010), which were freshly prepared and administered ad libitum to the drinking water in light protected bottles. Fecal pellets were collected immediately throughout the 86 days of the study and stored at −80°C until needed. Mice were monitored and weighed regularly and sacrificed at the conclusion of the study (day 86). See Supplementary Table 1 for more details on housing and samples.

### Virome sample processing

Two fecal pellets per sample were resuspended in 15 ml PBS buffer containing 0.01 M sodium sulfide and 10 mM EDTA for 30 min on ice. Samples were centrifuged 2 times at low speed (ThermoScientific Heraeus Multifuge 3SR) at 4°C for 30 min to remove bacteria and contaminating plant material. The resulting supernatants were sterile filtered and ultracentrifuged at 22,000 rpm (Beckman SW41 rotor) at 4°C for 2 h. Viral pellets were then resuspended in 200 µl Tris buffer (50 mM Tris, 5 mM CaCl_2_, 1.5 mM MgCl_2_, pH 8.0), from which 5 µl subsamples of isolated viruses were collected for morphological characterization by negative staining in 2% (w/v) aqueous uranyl acetate and visualized by transmission electron microscopy (TEM) (Technai Bio TWIN) at 80 kV with a magnification of 40,000–100,000. To the samples, 2 µl benzonase was added and incubated at 37°C for 2 h to remove remaining nucleic acid contamination.

To extract viral DNA and RNA, 22 µl of a 0.1 volume of 2M Tris–HCl (pH 8.5)/0.2 M EDTA, 10 µl of 0.5 M EDTA, and 268 µl of formamide were added to the sample and incubated at RT for 30 min. Subsequently, 1 µl of glycogen, and 1,024 µl of ethanol were added, and samples were mixed gently and incubated overnight at RT. The next morning, samples were centrifuged at 12,000×*g* at 4°C for 20 min, washed with 70% ethanol, and resuspended in 100 µl of TE buffer and 1 µl of mercaptoethanol, after which 10 µl of 10% SDS and 3 µl of Proteinase K were added and incubated for 20 min at 37°C and 15 min at 56°C. Then, 400 µl of DNA extraction buffer CTAB (100 mM Tris pH 8.0, 1.4 M NaCl, 20 mM EDTA, 2% CTAB) and 1 µl mercaptoethanol were added and samples were incubated at 56°C for 15 min. To the resulting supernatant, an equal volume of chloroform:isoamylalcohol (24:1) was added, and samples were centrifuged at 13,000 x *g* for 5 min. The supernatant was collected, to which 1 µl of glycogen, 10 µl mercaptoethanol, and a 0.7 volume of isopropanol were added and incubated overnight at −20°C. The next morning, samples were centrifuged at 13,000 x *g* at 4°C for 20 min, after which the supernatants were collected, washed with 500 µl of 70% ethanol, and stored at −80°C.

Following extraction of VLPs, ethanol was removed from samples and pellets were air-dried and resuspended in 20 µl of RNAse free filtered water. Amplification was performed using a modified Complete Whole Transcriptome Amplification Kit (WTA2) (Sigma-Aldrich) as described previously (Conceição-Neto *et al.* 2015). PCR products were then purified using the GenElute PCR Clean-Up Kit (Sigma-Aldrich). Samples were stored overnight at −20°C prior to library construction.

### Library construction

Libraries were generated using the NexteraXT kit (Illumina) (Conceição-Neto *et al.* 2015). After quantification, normalized pools of all samples were sequenced on an Illumina MiSeq using the 2 × 150 bp sequencing kit (Illumina). This Whole Genome Shotgun project has been deposited with the links to BioProject accession number PRJNA434045 in the NCBI BioProject database (https://www.ncbi.nlm.nih.gov/bioproject/?term=PRJNA434045) with BioSample accession numbers SAMN08534315 through SAMN08534344.

### Viral community composition

Nextera XT adapters were removed and sequence reads were trimmed from Illumina paired-end reads (2 × 150 bp) using Trimmomatic V.0.36 (Bolger *et al.* 2014). Trimmed and quality controlled reads of all samples were cross assembled using SPAdes V.3.1.10 (Bankevich *et al.* 2012) to generate a reference viral metagenome. Contigs were screened for contamination by using blastn against the NCBI nucleotide database (Pruitt *et al.* 2012). Contigs >90% identity and >50% of length were removed if no viral hallmark gene could be detected within the sequence. Sequence reads of all murine fecal viruses were assembled into 1,094,102 contigs. For our reference virome, we selected 4,767 contigs that were longer than 1,000 bp and had a coverage higher than 10. These contigs were selected and analyzed using blastx against the UniProt viral database including 5,571,160 viral sequences with an e-value cut off at 10^−5^ (Bateman *et al.* 2017). The reference virome had an average sequence length of 3,358 and a coverage of 80. Of these contigs, 48% were assigned to known viral sequences using the Uniprot viral database. Finally, the reference viral metagenome was further classified by VirSorter2 (Guo *et al.* 2021). To further refine phage config assignments, the tool vConTACT (Bolduc *et al.* 2017) was used to identify genomes into protein clusters (PCs) using BLASTP (Altschul *et al.* 1997) by comparing all proteins to the sequences, generating a similarity network. Groups of similar sequences were then clustered using the Markov clustering algorithm with an inflation value of 2 to create viral clusters (VCs). The relationship information identified from organized VCs and PCs were used to create a module profile, which can then be classified for taxonomic identification (Bolduc *et al.* 2017). The network was visualized with Cytoscape V.3.1.1 (Shannon *et al.* 2003), which places genomes sharing more PCs closer together (Supplementary Fig. 1).

In addition, all contigs were classified by the contig annotation tool CAT (Von Meijenfeldt et al. 2019) and submitted to Rapid Annotation using Subsystem Technology (RAST) to identify additional viral hallmark genes. Moreover, VirHostMatcherNet (Wang *et al.* 2020) and CAT (Von Meijenfeldt *et al.* 2019) were used to predict virus–prokaryote interaction. To reduce the impact of false positives we focused our viral community analysis only on contigs that were assigned as viral sequences based on VirSorter2, CAT, and RAST annotation. Contigs classified as a virus were used as OTUs representing the mice fecal viral community. This subset consisted of 614 contigs composed of approximately 94% dsDNA viruses, 5% ssDNA viruses, and 1% RNA viruses. Reads from each sample were then mapped separately against representative mice viral OTUs using the computer software Bowtie2 (Langmead and Salzberg 2012) and SAM tools (Li *et al.* 2009). The normalized coverage of each OTU was used as a proxy for the relative abundance of each virus per sample (Roux *et al.* 2016).

Viral community composition was analyzed using the computer software PRIMER V.7 (Clarke 1993; Anderson *et al.* 2008; Clarke and Gorley 2015), and abundance data were standardized and log + 1 transformed. Estimation of similarity between all samples was calculated by Bray–Curtis similarity and nonmetric multidimensional scaling analysis (MDS), and pairwise comparison of viral community composition between different treatment groups and time points was analyzed using a similarity test (ANOSIM global test) (Clarke 1993). SIMPER analysis was used to detect the most important viral OTUs that contribute to observed difference in community composition. These preselected OTUs were further analyzed by one-factor ANOVA followed by Tukey’s honest significant differences (HDS) test using the computer software IBM SPSS Statistics.

### Relationship between viral and bacterial community

To investigate the variability in the viral community that could be explained by the bacterial community composition, or vice versa, RELATE analysis (Anderson *et al.* 2008) in the computer software PRIMER V.7 (Clarke 1993; Clarke and Gorley 2015) was used. The analysis was based on the relative abundances of viral and bacterial OTUs. Raw bacterial FASTQ reads were obtained from our previous study (Moltzau Anderson *et al.* 2020) from EBI’s ENA under the Accession Number PRJEB21817 (http://www.ebi.ac.uk/ena/data/view/PRJEB21817). Viral and bacterial community datasets were standardized and log(*x* + 1) transformed. To investigate the variability of the bacterial community composition that could be explained by the viral community, we fitted the 29 most abundant viral OTUs with a minimum length of 10,000 bp to the relative abundance of bacterial OTUs using distance-based redundancy modeling (DISLM) with adjusted *R*^2^ selection criteria and forward selection procedure. Results were visualized with distance-based redundancy analysis (dbRDA) (Anderson 2001; McArdle and Anderson 2001).

## Results

### Presence of VLPs

**Fig. 2.**
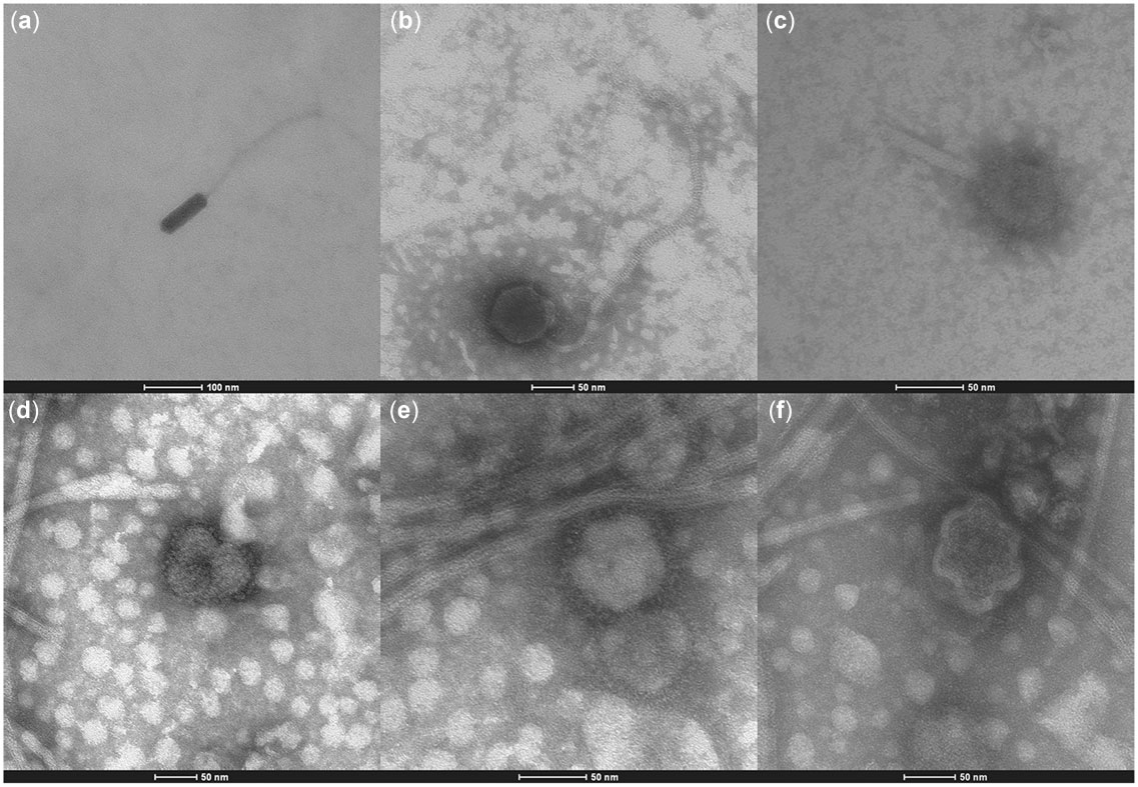
Transmission electron micrographs (TEM) of purified VLPs from murine feces negatively stained with 2% aqueous uranyl acetate. a–c) Bacteriophages from the family Siphoviridae and Myoviridae, respectively. d–f) Eukaryotic viruses with morphological similarity to PPR virus or MMTV virus.

The presence of VLPs in fecal samples was observed by TEM, which revealed morphologically distinct virions (Fig. 2). While ultracentrifugation may have damaged some, numerous diverse bacteriophages were present (Fig. 2a–c), and were distinguished by the structure of a head, or capsid, and in some cases a tail, although other phage morphologies exist beyond this structure (i.e. without a tail). The Myoviridae family morphology was present, with an icosahedral (20 sides) head and a contractile tail (Fig. 2c). The structure of a lambda-like phage (λ) displaying the Siphoviridae family morphology, which commonly infects *Escherichia coli*, was also observed (Fig. 2a and b). The protein head of the capsid is icosahedral (Fig. 2b) and elongated (Fig. 2a), containing the nucleic acid. The head is joined to a tail possessing a long thin tail fiber at its end (for host recognition). The tails are composed of a hollow tube, through which the nucleic acid passes into the host during infection. VLPs with morphological similarity to eukaryotic viruses, e.g. the *Peste des Petits Ruminants* (PPR) virus or the *Murine Mammary Tumor* virus (MMTV), were also observed (Fig. 2d–f).

### Reference murine fecal virome composition

The viral community was predominantly composed of dsDNA bacteriophages, consisting primarily of the order Caudovirales (71% of the viral contigs). To further refine dsDNA phage contig assignments, vConTACT was used to generate viral and PCs with known viral reference genomes for taxonomic identification (Supplementary Fig. 1). The network analysis depicted numerous clusters, including *Escherichia* phage, *Enterobacteria* phage, *Cellulophaga*, Microviridae, and *Lactococcus* phage, among others (Supplementary Fig. 1). Approximately 16% of the viral contigs were predicted by VirSorter2 as the dsDNA eukaryotic viruses Lavidaviridae and Nucleocytoplasmic large DNA viruses. To reduce the false positive detection of eukaryotic viruses, these contigs were compared by blastn and blastx to NCBI’s nonredundant protein database, most of which were found to have a high sequence similarity to prokaryotes rather than eukaryotes. These false positive eukaryotic viruses were removed. A few viral sequences were identified in murine feces that infect eukaryotes. One ssRNA virus of the family Retroviridae was found showing high sequence similarity on a nucleotide level to *Murine leukemia virus* (contig 3291). A dsRNA virus *Hordeum vulgare alphaendornavirus* (contigs 811, 1176, and 3773) of the family Endornaviridae was found, which is known to infect barley. Barley, among other plant material, was present in the dietary composition of the mouse feed. Other potential plant associated viruses that could be identified in this study were ssDNA viruses of the family Genomoviridae with high sequence similarity to *Gemycircularvirus* (contig 4238 and contig 2208).

### Incomplete recovery of the fecal viral community composition post-antibiotic perturbation

**Fig. 3.**
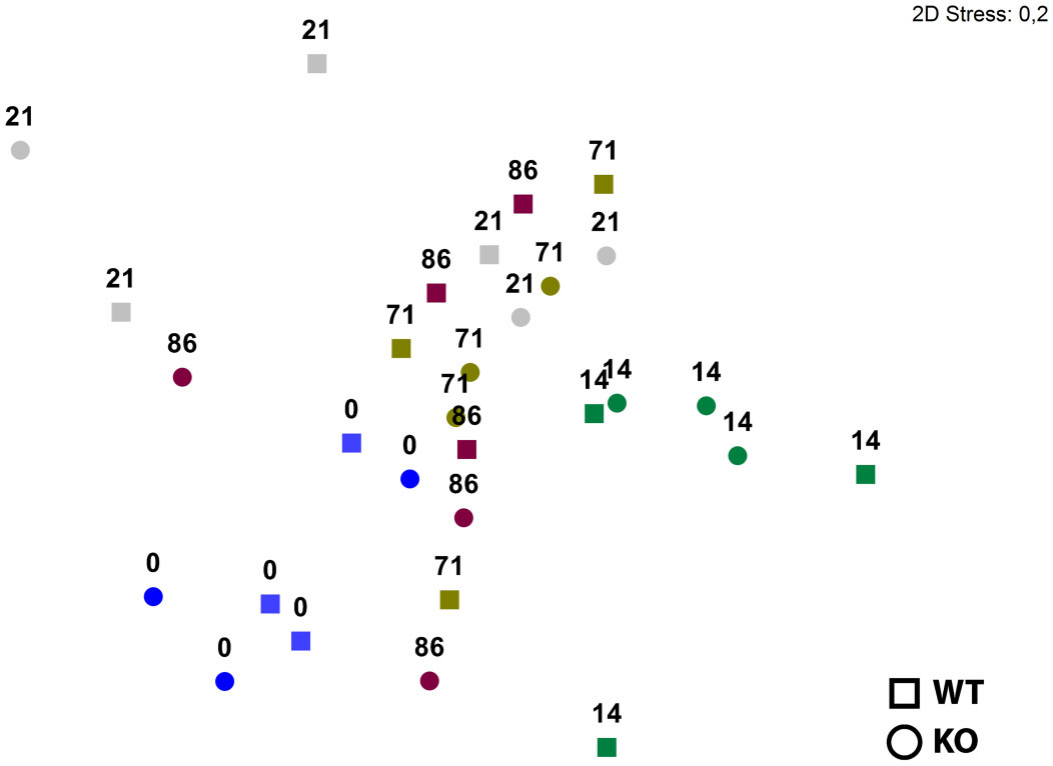
Nonmetric mutidimentional scaling (NMDS) analysis of the murine fecal viral community composition of *NOD2* KO (triangles, *n* = 3) and *C57BL/6J* WT (circles, *n* = 3). Analysis based on the Bray–Curtis similarity index of the relative abundance of viral OTUs at species level across time (day 0 = pre-treatment, day 14 = during treatment, days 21–86 = post-treatment).

MDS of the viral community based on viral OTU level demonstrated a clear clustering based on day (Fig. 3). Samples at day 0, prior to treatment, clustered together and communities underwent significant changes shifting after 14 days of antibiotic treatment (ANOSIM global test for test differences between time points: *R* statistics = 0.443, *P* = 0.001). Differences between the genotypes (WT vs *NOD2*-KO) could not be detected at any time point (ANOSIM global test for differences between time points: *R* statistic = −0.03, *P* = 0.753). Antibiotic treatment significantly changed the viral community composition (ANOSIM Pairwise Test; Supplementary Table 2). 20% of the average dissimilarity between day 0 and 14 was explained by the higher relative abundances of 4 phages infecting *Gammaproteobacteria* and a reduction of 2 phages infecting *Bacteroides* bacteria after antibiotic treatment (SIMPER analysis; Supplementary Table 3). We could confirm by ANOVA that *Escherichia* phages (contigs 14, 32, 186, and 3817) increased, whereas phages predicted to infect *Bacteroidetes*, such as Phage *apr34* (contig 52), and *Microvirus* (contig 996) were reduced after antibiotic treatment (*n* = 6, *P <* 0.01, one-way ANOVA, Tukey’s HDS). To further validate our findings, the 50 most important viral contigs were identified with the package Primer V.7 to generate a shade plot based on complete linkage clustering with Bray–Curtis similarity (Supplementary Fig. 2). In conjunction with our NMDS analysis (Fig. 3), samples did not cluster by genotype, but by day, where day 0 (pre-treatment) and day 14 (during the antibiotic treatment) clustered together respectively, after which a loose pattern of successive clustering occurred with days 21, 71, and 86 (Supplementary Fig. 2).

**Fig. 4.**
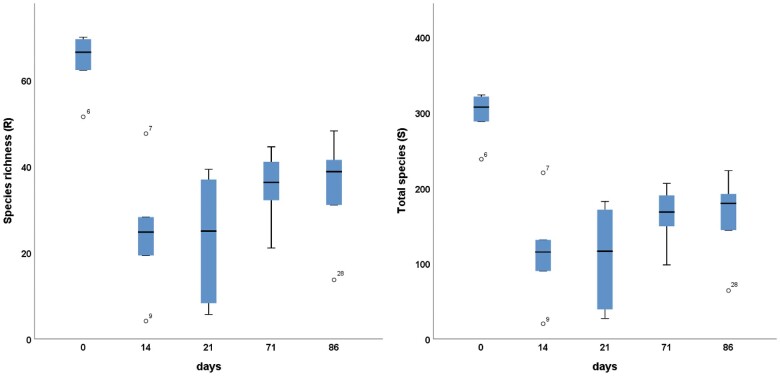
Viral diversity pre-antibiotic treatment (day 0) compared with post-treatment (post-day 14). day 0 had significantly higher viral diversity in both species richness (Margalef) and total species (*n* = 6, *F* = 13.525, *P <* 0.001, one-way ANOVA, Tukey’s HDS).

The community compositional trajectory shifted toward recovery with increasing time by clustering more closely with day 0 (i.e. developing toward a community composition similar to the initial community prior to the antibiotic treatment) (Fig. 3). However, viral community composition at day 86 did not fully recover and remained significantly different from day 0 with an average dissimilarity of 86.64 (ANOSIM Pairwise Test for differences between days 0 and 86: *R* statistics = 0.581, *P* = 0.002) indicative of an incomplete recovery (Fig. 3; Supplementary Fig. 2). Of this dissimilarity, 20% could be explained by as few as 6 viral OTUs, of which *Microvirus* (contig 935) and Phage *apr34* (contig 52) were highly reduced after the antibiotic perturbation and could not be detected at the end of the study (day 86) (Supplementary Table 4). Moreover, comparing viral diversity pre-antibiotic treatment compared with post-treatment, day 0 (pre-treatment) had a significantly higher viral diversity in both total species (*n* = 6, *F* = 13.525, *P <* 0.001, one-way ANOVA, Tukey’s HDS) and species richness (Margalef) (*n* = 6, *F* = 13.527, *P <* 0.001, one-way ANOVA, Tukey’s HDS) (Fig. 4).

### High interindividual variation of prokaryote viral community composition

**Fig. 5.**
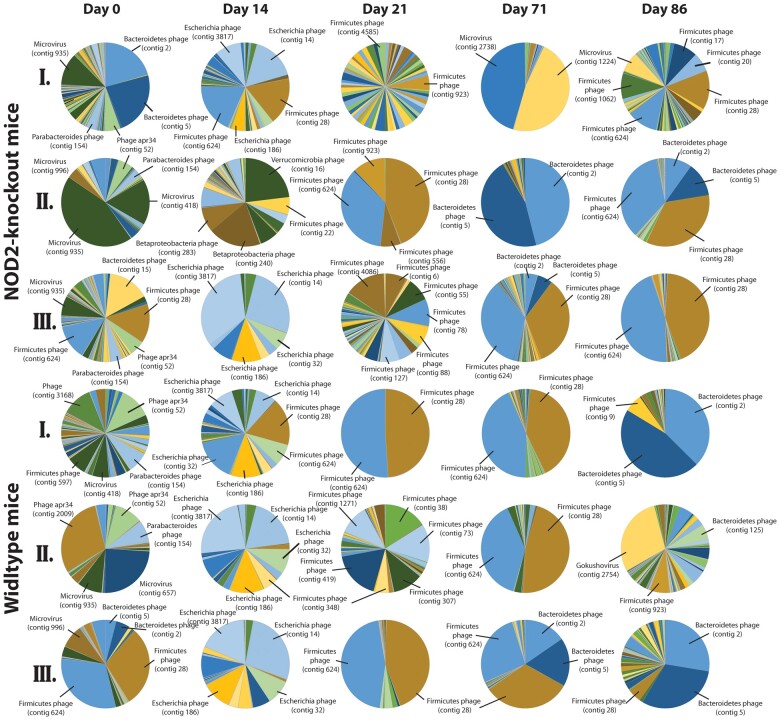
Prokaryotic viral community composition in *NOD2* KO (*n* = 3) and *C57BL/6J* WT (*n* = 3) mice across time (day 0 = pre-treatment, day 14 = antibiotic treatment, days 21–86 = post-treatment). Pie charts illustrate the relative abundance of prokaryotic viral contigs based on the mapping of individual sequence reads to the murine fecal reference virome which was generated by cross assembly of all sequence reads. Roman numbers represent individual mice.

**Fig. 6.**
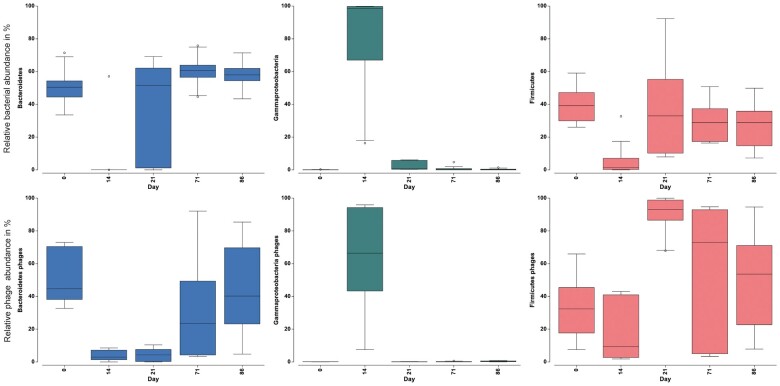
Relative abundance (%) of *Bacteroidetes*, *Firmicutes*, and *Gammaproteobacteria* phages, compared with the relative abundance (%) of the corresponding host bacteria. Phages were grouped by their bacterial host predicted by VirHostMatcherNet and CAT taxonomy. Time is represented in days (day 0 = pre-treatment, day 14 = antibiotic treatment, days 21–86 = post-treatment) (error bars = ±2 SE, *n* = 3).

Fecal bacteriophages were diverse and variable in their relative abundance between different individual mice. Prior to antibiotics (day 0) dominant phages were Microviridae, Phage *apr34* and other *Bacteroidetes* and *Firmicutes* infecting phages (Fig. 5). Antibiotic perturbation strongly affected phage composition shifting to an *Escherichia* phage dominated system. Within 1 week of antibiotic cessation the viral community composition demonstrated huge variability. No clear pattern of recovery over time or between the genotypes could be observed, with high interindividual variation of viral community composition present throughout (Fig. 5). The analysis on an OTU level indicated shifts in *Gammaproteobacteria*, *Firmicutes*, and *Bacteroidetes* phages. To determine whether these shifts in the phage population were significant, we used VirHostMatcherNet and CAT taxonomy for bacterial host prediction. All phages were grouped based on their bacterial host prediction at a higher phylogenetic level (i.e. *Gammaproteobacteria*, *Firmicutes*, and *Bacteroidetes*) (Fig. 6). Prior to the antibiotic treatment, the phage population was dominated by equal portions of *Bacteroidetes* and *Firmicutes* phages. Large changes within the community composition occurred during antibiotic treatment (day 14), which was distinct from pre-treatment at day 0 (Figs. 3 and 5). During this time, the phage community was dominated by *Gammaproteobacteria* phages (Fig. 6).

After antibiotic perturbation, *Bacteroidetes* phages were significantly reduced from 44.8% to 5.3% at day 21 (*n* = 6, *F* = 3.475, *P* = 0.025, one-way ANOVA, Tukey’s HDS). The relative abundances of Firmicutes phages were only slightly affected by antibiotic perturbation from 40% to 17.8% at the end of antibiotic treatment (day 14), and not significantly affected compared with pre-antibiotic treatment. During the recovery period (post day 14), the relative abundance of *Gammaproteobacteria* phages was reduced, while *Firmicutes* phages recovered rapidly and reached higher relative abundance compared with pre-antibiotic perturbation at day 21 (*n* = 6, *F* = 5.78, *P* = 0.013, one-way ANOVA, Tukey’s HDS). This was in contradiction to observed shifts in the bacterial communities, which featured higher relative abundances of *Bacteroidetes* bacteria compared with *Firmicutes* bacteria at day 71 (*n* = 6, *F* = 20.4, *P* = 0.001, one-way ANOVA, Tukey’s HDS) and day 86 (*n* = 6, *F* = 18.3, *P* = 0.002, one-way ANOVA, Tukey’s HDS). In contrast to *Firmicutes* phages, recovery of *Bacteroidetes* phages was delayed and only detectable from day 71. Interestingly, *Bacteriodetes* and *Firmicutes* bacteria were differently affected by the antibiotic perturbation showing an almost total eradication of *Bacteroidetes* after 12 days of antibiotic treatment, while *Firmicutes* bacterial population still reached 7% relative abundance at day 14.

**Fig. 7.**
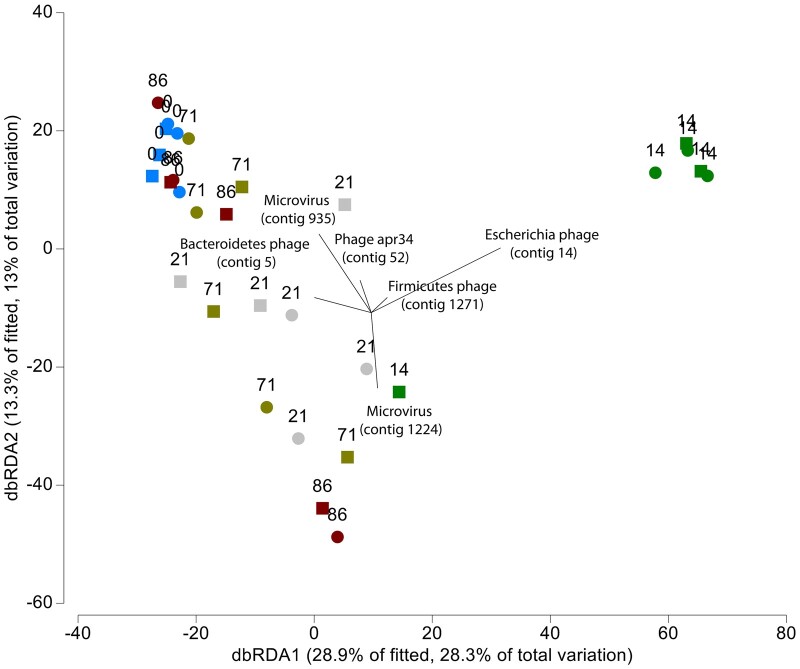
Constrained dbRDA plot of the murine fecal viral community composition of *NOD2* KO (triangles, *n* = 3) and *C57BL/6J* WT (circles, *n* = 3) across time fitted to the fecal bacterial community composition in DistLM sequential tests with *R*^2^ selection criteria and forward selection procedure. Lengths of vector overlays indicate the relative influences of related predictor variables. Time is represented in days (day 0 = pre-treatment, day 14 = antibiotic treatment, days 21–86 = post-treatment).

Changes in the compositional shifts of both bacterial and viral communities were correlated, where changes in the bacterial community composition (based on an OTU level) occurred in a similar direction and magnitude as the compositional shifts of the viral community (RELATE, Rho = 0.416, *P* = 0.001, 999 permutations). Time after the antibiotic perturbation (recovery period) was the main factor in both groups. Using distance-based modeling (DISLM) of the 29 most important phages, the relative abundance of *Escherichia phage* (contig 14) was found to be higher from the antibiotic perturbation and explained 25.5% of the variation in the bacterial community (Fig. 7). Together with 4 other bacteriophages, the model could explain up to 49% of the bacterial variation (Fig. 7; Supplementary Table 5).

## Discussion

Investigations of the gut microbiota have largely focused on the bacteriome, compared with the virome, and RNA viruses were not included in most studies. To our knowledge, resilience properties of the virome in IBD post an antibiotic perturbation have never been explored. Here, we longitudinally characterized the dynamic murine gut virome in wild-type and *NOD2* KO mice by collecting fecal samples prior to, during, and post-treatment. Contrary to our expectations, there were no significant differences in the viral community between the genotypes. Fecal samples are widely used in microbial community analysis, and while luminal content or mucosal samples may have uncovered distinct differences associated with the *NOD2* phenotype and activity, this would have required sacrificing mice at different time points, thereby losing the power of intraindividual temporal effects. Nevertheless, we demonstrated that the murine fecal virome is morphologically and genetically diverse, including viruses infecting the host (eukaryotes, i.e. Retroviridae, *Murine leukemia virus*, etc.), viruses infecting prokaryotes (bacteriophages, i.e. Caudovirales), and viruses infecting neither of them (plant viruses, i.e. *Hordeum vulgare alphaendornavirus*).

The murine gut virome also shares several characteristics to the human gut virome. Firstly, the murine gut virome was highly variable among individuals. This high interindividual viral diversity has also been previously reported in the human gut virome (Reyes *et al.* 2010; Norman *et al.* 2015). Furthermore, similarly to the human gut virome, the murine gut virome was established to contain a large diversity of primarily bacteriophages, in addition to a much lower diversity of eukaryotic viruses (Reyes *et al.* 2010; Minot *et al.* 2011, 2013). Additionally, consistent with previous reports, the most abundant viral taxa identified were bacteriophages of the order Caudovirales (i.e. *Cellulophaga* phage) and the family Microviridae (i.e. *Parabacteroides* phage) (Reyes *et al.* 2010; Minot *et al.* 2011; Norman *et al.* 2015).

RNA viruses associated with murine feces were also identified, and ultimately, only these RNA viruses could be verified as eukaryotic viruses. Of these eukaryotic viruses, some were identified as having a plant host (i.e. *Hordeum vulgare alphaendornavirus* known to infect barley). Plant viral sequences likely reflect the omnivorous diet of these mice, which included barley, wheat, soybean products, oat hulls, and sugar beet pulp, and we speculate that diet plays a significant role in the composition of the gut eukaryotic virome. Interestingly, the detection of these viruses was predominantly from day 14, which had the lowest phage diversity. We further speculate that these plant viruses are always present, but could not be detected because of the greater phage community. Most studies to date have not reported the presence of large eukaryotic viruses as a result of filtering methods during isolation and extraction (Conceição-Neto *et al.* 2015). For instance, although filtering methods efficiently remove bacteria, filters have also been shown to remove more than 99% of *Mimivirus* and 90% of herpes viruses (Vijay-Kumar *et al.* 2010). It is possible that these viruses are present more often than originally considered, though it remains to be seen what role they may play within the host. It should also be mentioned that there is an urgent need to develop improved references for characterizing the virome, as evidenced by the large percentage of sequences that were unclassified in the assembled reference, yet originated from viral fractions. This may have caused some viral contigs to be discarded, specifically prophages in bacterial genomes. However, only contigs without known viral hallmark genes were removed from our analysis. Moreover, fragmentation of a viral genome will cause some parts of the sequence to be removed (i.e. those not containing viral specific genes), but will retain other parts of the same viral genome that do (e.g. containing phage tail fibers or shaft proteins), ultimately keeping it in the reference virome. The resulting sequencing catalog generated from this study is composed of nearly full genomes of high quality, serving as an important reference for future virome studies.

To induce a significant perturbation, we used the broad-spectrum antibiotic combination composed of ampicillin, vancomycin, neomycin, and metronidazole based on a well-established commensal depletion protocol (Fagarasan *et al.* 2002; Rakoff-Nahoum *et al.* 2004; Vijay-Kumar *et al.* 2010). This caused a significant change on the viral community composition during and after the antibiotic perturbation. Although antibiotics do not directly target viruses, our results demonstrated significant changes in the bacterial community, which coincided with shifts of the fecal bacteriophage community. Decidedly, how the microbiome responds to antibiotics is altered by both the initial state of the microbiome prior to the perturbation (i.e. host factors, microbial species, functional diversity) and by the strength and magnitude of the perturbation (i.e. antibiotic dose, duration, spectrum) (Sommer *et al.* 2017). Prior to antibiotic perturbation, the phage population was dominated by equal proportions of *Bacteroidetes* phages and *Firmicutes* phages, reflecting the bacterial community which was dominated by *Bacteroidetes* and *Firmicutes*. During antibiotic treatment, *Bacteroidetes* phages were largely reduced and replaced by *Gammaproteobacteria* phages. It is possible that the outgrowth of *E. coli*/*Shigella* during antibiotic treatment led to the bloom of their respective bacteriophage (i.e. *Escherichia shigella* phage) as expected in Lotka-Volterra/Kill-the-Winner dynamics (Rodriguez-Valera *et al.* 2009; Mirzaei and Maurice 2017). This effect is caused by the use of vancomycin as a component of the antibiotic cocktail and has been previously shown to promote the expansion of *Proteobacteria* (Carvalho *et al.* 2012; Rooks *et al.* 2014) including Enterobacteriaceae and *E.coli* (Rooks *et al.* 2014). Notably, enrichment of Enterobacteriaceae is an important indicator of dysbiosis. Interestingly, numerous studies have demonstrated a significant association between the *NOD2* risk allele and the increase in relative abundance of Enterobacteriaceae (Garrett *et al.* 2007; Gevers *et al.* 2014; Knights *et al.* 2014). However, contrary to a previous study in humans, no unique changes occurred in the bacteriophage community specific to the *NOD2* KO mice (Norman *et al.* 2015). After the antibiotic perturbation, compositional shifts in the murine bacterial and viral communities were significantly correlated, where changes in the communities occurred in a similar direction and magnitude. *Gammaproteobacteria* phages were reduced, whereas an increase in *Firmicutes* phages occurred and remained until the end of the study. These changes in the phage community were also reflected in the bacterial community through an increase in the phyla *Firmicutes*. On the other hand, while *Bacteroidetes* phages did increase after day 21, their relative abundance remained low compared with day 0. This was in contradiction to observed shifts in the bacterial community, which featured higher relative abundances of *Bacteroidetes* bacteria compared with *Firmicutes* bacteria at days 71 and 86. It is possible that the loss of *Croceibacter* phage, which disappeared during the antibiotic perturbation, may have been a regulator of *Bacteroidetes* bacteria, further revealing that virus–bacteria community dynamics of the gut are complex. However, due to limited fecal material from the initial study design (Moltzau Anderson *et al.* 2020), we lacked timepoints between day 21 and 71, which restricts insights into the exact timing of events. Nonetheless, the identification of *Firmicutes* and *Bacteroidetes* phages associated with their bacterial phyla (i.e. *Firmicutes* and *Bacteroidetes*) both prior to the perturbation and at the conclusion of the study, suggests an important role in the virus-bacterial dynamics of these communities in maintaining host health. Ultimately, the viral community had an impaired recovery, though the community appeared to be reapproaching a structure similar to day 0 (pre-treatment).

Our work extends previous observations from experiments studying recovery and resilience of fecal microbial communities across time (Dethlefsen *et al.* 2008; David *et al.* 2014; Heinsen *et al.* 2015; Moltzau Anderson *et al.* 2020). Antibiotic perturbation caused substantial shifts in the murine fecal viral community. After antibiotic treatment we observed a delayed and incomplete recovery of the fecal virome, which was characterized by high interindividual variation. The absence of functional *NOD2* signaling had no additional effect in our experiment. Our results indicated that dynamics of bacteriophage abundances during and after antibiotic treatment is tightly linked to bacterial community shifts, and their loss might contribute to a disturbed microbiome. Our results highlight the importance of better understanding factors contributing to resilience of the intestinal ecosystem in health and disease. Targeting the virome may therefore represent a novel approach in microbiota-targeted therapies, which are currently explored in a variety of intestinal disorders, from IBD to colorectal cancer.

## Supplementary Material

jkac293_Supplementary_Data

## Data Availability

The viral and bacterial datasets are publicly available from NCBI’s BioProject under the accession number PRJNA434045 and EBI’s ENA under the accession number PRJEB21817. For more details, see *Materials and methods* section. Supplemental material is available at G3 online.
