# Supplementary material for: Impact of antibiotic perturbation on fecal viral communities in mice
Source: G3 (Bethesda). 2022 Nov 22;13(1):jkac293. doi: 10.1093/g3journal/jkac293 (PMC9836353; doi:10.1093/g3journal/jkac293)
Supplement: jkac293_Supplementary_Data [file jkac293_supplementary_data.zip › Suppl/Supplemental_Figure_Legends_G3-2022-403621.docx]

**Supplementary Figure Legends**

**Figure S1.** vConTACT network analysis. Clustering of mouse fecal dsDNA viral contigs identified by VirSorter (olive green) with known virus reference genomes (teal green).

**Figure S2.** Shade plot of mouse fecal viral community composition illustrating the relative abundance of a subset of the 50 most important viral contigs (left). Dendrograms are based on complete linkage clustering with Bray-Curtis similarity. Day 0 (pre-treatment), Day 14 (during treatment), and Days 21, 71 and 86 (recovery period); NOD2 KO n = 3, C57BL/6J WT n = 3.
