# Supplementary figures and images for: Impact of antibiotic perturbation on fecal viral communities in mice
Source: G3 (Bethesda). 2022 Nov 22;13(1):jkac293. doi: 10.1093/g3journal/jkac293 (PMC9836353; doi:10.1093/g3journal/jkac293)

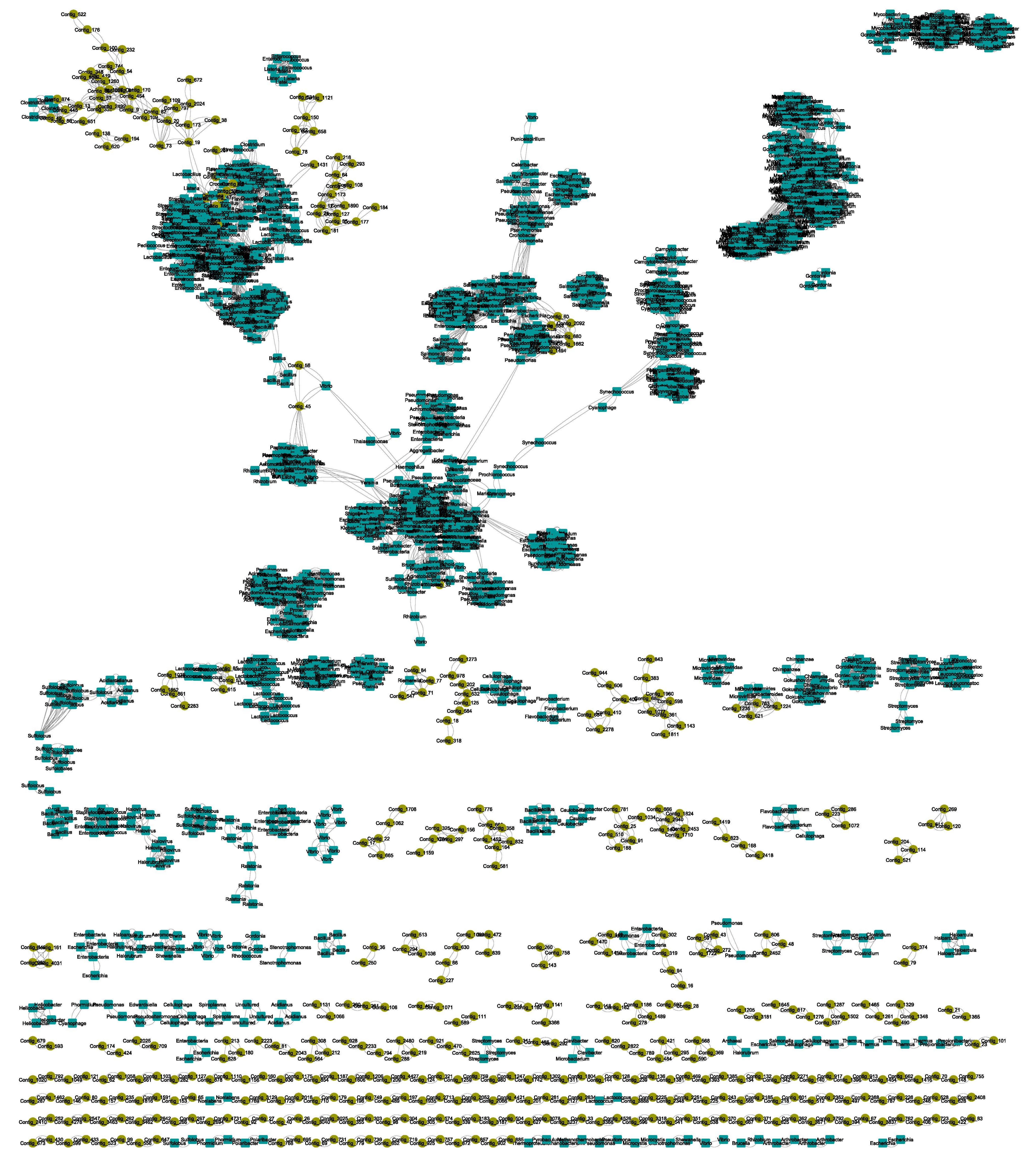

Supplement: jkac293_Supplementary_Data [file jkac293_supplementary_data.zip › Suppl/Supplemental_Figure_S1_G3-2022-403621.pdf]

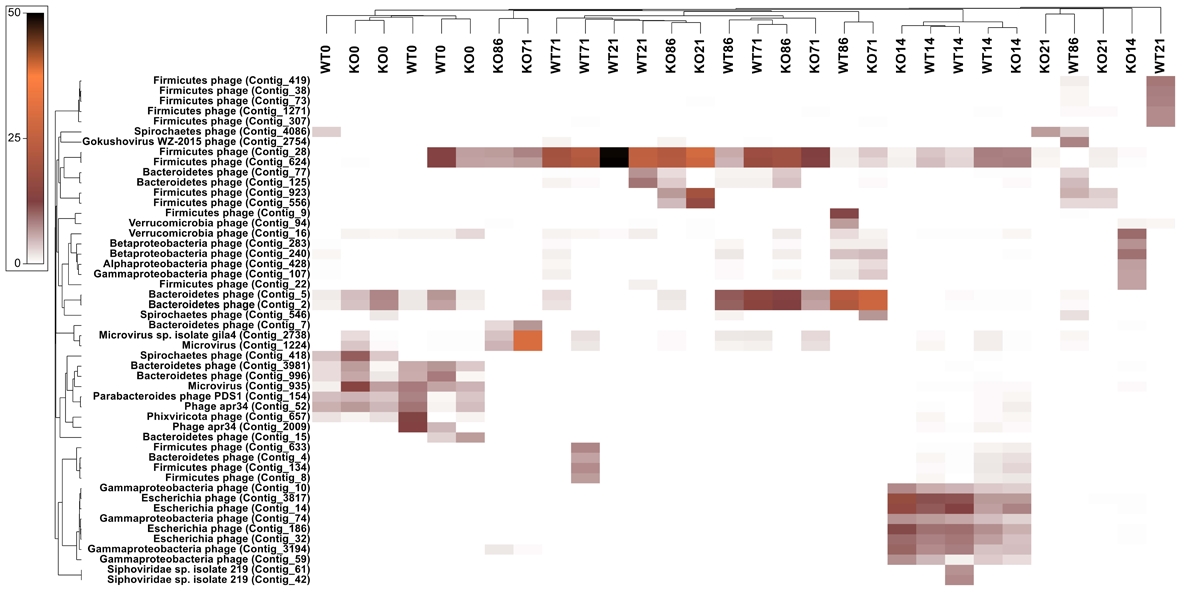

Supplement: jkac293_Supplementary_Data [file jkac293_supplementary_data.zip › Suppl/Supplemental_Figure_S2_G3-2022-403621.tif]
